# Comparison of Five Parathyroid Scintigraphic Protocols

**DOI:** 10.1155/2013/921260

**Published:** 2013-01-21

**Authors:** Virpi Tunninen, Pekka Varjo, Jukka Schildt, Aapo Ahonen, Tomi Kauppinen, Irina Lisinen, Anu Holm, Hannu Eskola, Marko Seppänen

**Affiliations:** ^1^Department of Nuclear Medicine, Satakunta Central Hospital, Sairaalantie 3, 28500 Pori, Finland; ^2^Department of Clinical Physiology and Nuclear Medicine, Helsinki University Central Hospital, HUS, P.O. Box 340, 00029 Helsinki, Finland; ^3^HUS Medical Imaging Center, Helsinki University Central Hospital, P.O. Box 340, 00029 Helsinki, Finland; ^4^Turku PET Centre, Turku University Hospital, P.O. Box 52, 20521 Turku, Finland; ^5^Department of Biomedical Engineering, Tampere University of Technology, P.O. Box 527, 33101 Tampere, Finland; ^6^Department of Clinical Physiology and Nuclear Medicine, Turku University Hospital, P.O. Box 52, 20521 Turku, Finland

## Abstract

*Objectives*. We compared five parathyroid scintigraphy protocols in patients with primary (pHPT) and secondary hyperparathyroidism (sHPT) and studied the interobserver agreement. The dual-tracer method (^99m^Tc-sestamibi/^123^I) was used with three acquisition techniques (parallel-hole planar, pinhole planar, and SPECT/CT). The single-tracer method (^99m^Tc-sestamibi) was used with two acquisition techniques (double-phase parallel-hole planar, and SPECT/CT). Thus five protocols were used, resulting in five sets of images. *Materials and Methods*. Image sets of 51 patients were retrospectively graded by four experienced nuclear medicine physicians. The final study group consisted of 24 patients (21 pHPT, 3 sHPT) who had been operated upon. Surgical and histopathologic findings were used as the standard of comparison. *Results*. Thirty abnormal parathyroid glands were found in 24 patients. The sensitivities of the dual-tracer method (76.7–80.0%) were similar (*P* = 1.0). The sensitivities of the single-tracer method (13.3–31.6%) were similar (*P* = 0.625). All differences in sensitivity between these two methods were statistically significant (*P* < 0.012). The interobserver agreement was good. *Conclusion*. This study indicates that any dual-tracer protocol with ^99m^Tc-sestamibi and ^123^I is superior for enlarged parathyroid gland localization when compared with single-tracer protocols using ^99m^Tc-sestamibi alone. The parathyroid scintigraphy was found to be independent of the reporter.

## 1. Introduction

^99m^Tc-methoxyisobutylisonitrile (^99m^Tc-sestamibi), first introduced by Coakley and coworkers as a parathyroid imaging agent in 1989 [1], is the imaging agent of choice for parathyroid scintigraphy (PS) [2]. Unfortunately, ^99m^Tc-sestamibi is not a specific tracer for parathyroid tissue but is taken up by adjacent thyroid tissue. This problem can be overcome by using either a single-tracer (double phase) or a dual-tracer method.

In the single tracer method, it is assumed that thyroid and parathyroid tissues have different washout kinetics for ^99m^Tc-sestamibi [3]. By acquiring images in the early and late phases, the focally increasing uptake will reveal hyperfunctioning parathyroid tissue. In the dual-tracer method, ^99m^Tc-sestamibi is used combined with ^123^I or ^99m^Tc-pertechnetate, which are taken up by the thyroid gland only. Subtracting the thyroid image from the ^99m^Tc-sestamibi image provides visualization of the parathyroid tissue alone [4].

With both single-tracer and dual-tracer methods, several acquisition techniques can be used (i.e., planar acquisitions with parallel-hole or pinhole collimators and SPECT or SPECT/CT), and several choices can be made about the settings used for each technique (e.g., matrix size, energy settings, and acquisition time). There are several studies that provide comparisons between the different imaging methods or techniques, although there is little evidence supporting the superiority of one over another, resulting in the use of various study protocols today [5, 6].

We have previously shown that there is significant variability in the current practice of PS in Finland [7]. This is also true in other countries, with reported sensitivities for localizing abnormal parathyroid tissue ranging from 34% to 100% [8].

As a result of our previous study, the clinical protocol of parathyroid scintigraphy in Satakunta central hospital was changed in June 2010. Pinhole and SPECT/CT acquisition techniques were included to increase the sensitivity of the study. Additional late phase imaging was also included to benefit from the double phase method as well.

The goal of this study was to compare the sensitivity and specificity of a single-tracer method and a dual-tracer method with various acquisition techniques. The dual-tracer method (^99m^Tc-sestamibi/^123^I) was used with three acquisition techniques (parallel-hole planar, pinhole planar, and SPECT/CT). The single-tracer method (^99m^Tc-sestamibi) was used with two acquisition techniques (double phase parallel-hole planar, and SPECT/CT). In addition, the agreement between the findings of four experienced nuclear medicine physicians was studied.

## 2. Methods

### 2.1. Patients

This was a retrospective single-center study of fifty-one patients referred for PS between June 2010 and February 2011 in Satakunta Central Hospital, Finland. Patient data were included if there was biochemical evidence of hyperparathyroidism, if scintigraphy was requested for preoperative tumor localization, and if the patient proceeded to surgery. Histopathological finding was used as the gold standard. The final study group consisted of 6 men and 18 women with a mean age of 62.3 years (range, 32.1–86.8 years). Twenty-one patients had pHPT. Preoperative plasma intact parathyroid hormone (iPTH) values ranged from 69 ng/L to 277 ng/L (mean 190 ng/L, normal values 10–65 ng/L), and values for preoperative serum calcium (Ca) ranged from 1.37 mmol/L to 1.73 mmol/L (mean 1.48 mmol/L, normal values 1.16–1.3 mmol/L). Three patients had sHPT due to renal failure. Preoperative iPTH values ranged from 210 ng/L to 400 ng/L (mean 380 ng/L), values for preoperative Ca ranged from 1.21 mmol/L to 1.8 mmol/L (mean 1.45 mmol/L). Twenty-seven patients did not proceed to surgery for a variety of reasons (patient condition, death, and other illnesses). This study was exempt from institutional review board approval according to Finnish legislation. Informed consent was waived.

### 2.2. Imaging: Doses and Acquisition

**Figure 1:**
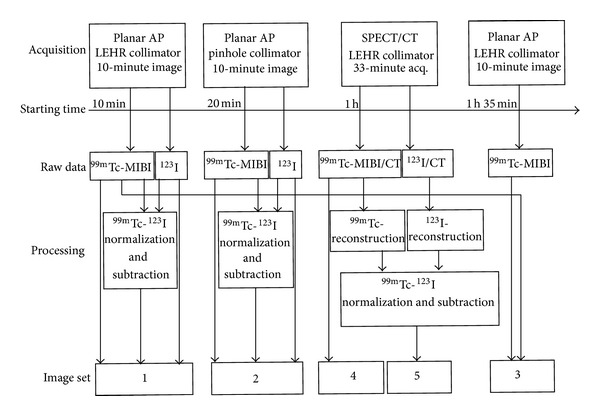
The orders and the timings of the acquisitions and the resulting image sets. Set 1: ^99m^Tc-sestamibi, ^123^I, and subtraction images with parallel-hole collimator. Set 2: ^99m^Tc-sestamibi, ^123^I, and subtraction images with pinhole collimator. Set 3: ^99m^Tc-sestamibi double phase images with parallel-hole collimator. Set 4: ^99m^Tc-sestamibi SPECT/CT. Set 5: ^99m^Tc-sestamibi, ^123^I, and subtraction SPECT/CT images.

Patients received 20 MBq of ^123^I (MAP Medical Technologies) intravenously. Two hours later, 550 MBq of ^99m^Tc-sestamibi (Mallinckrodt Medical B.V.) was injected intravenously. Ten minutes after the ^99m^Tc-sestamibi administration, imaging was started. Five different image sets were acquired. The order and the timing (after the injection or ^99m^Tc-sestamibi) of the acquisitions and the resulting image sets are presented in Figure 1.

First, a static 10-minute anterior image of the neck and chest was acquired using a low-energy, high-resolution, parallel-hole collimator (LEHR) (256 × 256 matrix; 1.85x zoom). Next, a static 10-minute anterior image of the neck was acquired from a distance of 10 cm from the patient's skin using a 5 mm diameter pinhole collimator (256 × 256 matrix; 2.19x zoom). Acquisitions were performed with the same dual-head gamma camera (Skylight; Philips). All data were collected in dual-energy windows. The ^99m^Tc window was centered at 140 keV and had a 10% width (range, 133–147 keV). The ^123^I window was centered at 159 keV and had a 10% width (range, 151–167 keV). Narrow windows were used to minimize crosstalk between isotopes.

One hour after the ^99m^Tc-sestamibi injection, the SPECT/CT acquisition was started (Symbia T; Siemens). SPECT data were acquired in a step-and-shoot sequence with a noncircular orbit (180° detector configuration; low-energy, parallel-hole, high-resolution collimators; 128 × 128 matrix; 4.8 mm pixel size; 48 views for each detector (3,75° per projection); 33 s/projection; total scan time, 32 min). All data were collected in dual-energy windows. The ^99m^Tc window was centered at 140 keV and had a 15% width (range, 129.5–150.5 keV). The ^123^I window was placed with a 4% offset above 159 keV and had a 15% width (range, 153.4–177.3 keV). The 4% offset was used to minimize the spillover of the ^99m^Tc photopeak into the ^123^I photopeak. After the SPECT acquisition was complete, the patient remained still on the table for the CT acquisition. A topogram scout scan (130 kVp, 30 mA, anterior view) was performed first, and limits for the CT acquisition were set (from the neck to the diaphragm). Then, a helical CT scan was performed (130 kVp, 2 × 2.5 mm collimation, 0.8 s rotation time, 1.5 pitch). The dose was controlled by tube-current modulation (CARE Dose AEC+DOM; Siemens), with the reference exposure set to 30 mAs.

Finally, a static 10-minute anterior image of the neck and chest was acquired using a low-energy, high-resolution collimator with a Siemens Symbia T-gamma camera (256 × 256 matrix; 1.85x zoom (32.2 cm field)). The same energy settings as those in the SPECT acquisition were used. Eleven of the patients did not complete this final image due to limited camera time or patient-related reasons. A ^99m^Tc intrinsic flood was used for both energy windows in both cameras. It was verified that the image-field uniformity was acceptable for all energy windows used.

### 2.3. Image Processing

All planar images were analyzed in a Hermes workstation (Hermes Medical Solutions). For dual-tracer images, a normalization factor (NF) was defined as the ratio of the thyroid maximum pixel counts in the ^123^I and ^99m^Tc-sestamibi images. Gradient subtraction images were created by multiplying the ^99m^Tc-sestamibi image with 10 successive NFs (with 20% steps from 20% to 200% of the original NF), and the ^123^I image was subtracted from each normalized ^99m^Tc-sestamibi image, resulting in 10 subtraction images to avoid oversubtraction [9, 10]. The final image sets consisted of ^99m^Tc-sestamibi and ^123^I images and gradient subtraction images (image set 1 acquired with LEHR, image set 2 with pinhole). ^99m^Tc-sestamibi early- and late-phase images were displayed side-by-side on the Hermes workstation (image set 3).

SPECT images were reconstructed on the Siemens Syngo workstation (Siemens) using the FLASH 3D algorithm (8 iterations, 8 subsets, Gaussian 9.00 filter). No scatter correction was used. The initial NF was defined as the ratio of the corresponding thyroid maximum voxel counts in ^99m^Tc-sestamibi and ^123^I SPECT data. ^123^I SPECT data were multiplied by NF to create normalized ^123^I SPECT data, which were then subtracted from ^99m^Tc-sestamibi SPECT data to create the subtraction SPECT dataset, as described by Neumann and coworkers [4]. The NF was adjusted until the subtraction SPECT images were subjectively satisfactory. CT data were reconstructed on the Siemens Syngo workstation (Siemens) for attenuation correction using the B08s kernel, and for fusion display purposes with a B40s medium kernel. The CT images were downsampled to match the SPECT image matrix and converted from Hounsfield units into effective attenuation values at 140 keV (^99m^Tc) and 159 keV (^123^I). The final image sets consisted of ^99m^Tc-sestamibi SPECT/CT data (image set 4) and ^123^I, ^99m^Tc-sestamibi, and subtraction SPECT/CT data (image set 5). The accuracy of the SPECT/CT data coregistration was checked. All image processing was performed by an experienced medical physicist.

### 2.4. Image Interpretation

All patient image datasets were anonymized before review by four experienced nuclear medicine physicians, who were blinded to all patient-related information. Five image sets (Figure 1) were reviewed. Datasets 1, 2, and 3 were read in separate reading sessions. Image sets 4 and 5 were reviewed in a single session in this order, with the physician being aware that the datasets belonged to the same patient.

Each quadrant in relation to the thyroid gland (right upper, right lower, left upper, and left lower) was classified on a 3-point scale (0 = negative, 1 = uncertain, and 2 = positive). The image review criteria for positive finding were as follows: (a) for image sets 1 and 2: clear abnormal residual activity on the planar subtraction images, (b) for image set 3: focally increased uptake that persisted or increased in intensity from early to late images, (c) for image set 4: focally increased uptake outside the normal ^99m^Tc-sestamibi biodistribution that had an anatomic correlation in the CT images, and (d) for image set 5: clear abnormal residual activity on the subtraction SPECT images that had an anatomic correlation in the CT images.

### 2.5. Surgery and Histologic Analysis

All patients were operated upon by an endocrine surgeon using an open technique. The surgeon was aware of all initial scintigraphic results prior to surgery. All glands were not identified for all patients. Postoperative iPTH and Ca values were reviewed to confirm surgery success. The mean interval between scintigraphy and surgery was 181 days (range, 29–457 days). A histopathological analysis was performed for all excised tissue.

### 2.6. Data Analysis

To estimate the sensitivity, specificity, and accuracy for the localization for each image set, scores of 0-1 were considered negative and scores of 2 were considered positive. Findings were classified as true positive, false positive, true negative, or false negative with histologic analysis as the reference standard. For each patient, four scores, one for each quadrant, were assigned. The false-positive image rate was defined as the ratio of false positives to the sum of true positives plus false positives [11].

### 2.7. Statistical Methods

The sensitivity, specificity, and accuracy of each image set were calculated for each physician separately. A McNemar test was performed to compare the sensitivities, specificities, and accuracies between the image sets. The results from physician 1 were chosen when comparing the image sets, as he had the most experience with the imaging methods and techniques used. The Mann-Whitney *U* nonparametric test was used to compare the size of the visualized and nonvisualized glands. A McNemar test was also used to analyze the accuracy of the different physicians. The differences for each method/technique were analyzed separately. *κ* coefficients were used to quantify the agreement between the results from the four physicians. Positive kappa values within the ranges of 0.01–0.20, 0.21–0.4, 0.41–0.60, 0.61–0.80, and 0.81–1.00 were interpreted as “very weak,” “weak,” “medium,” “good,” and “very good” agreement, respectively [12]. A *P* value <0.05 was considered statistically significant. Statistical analyses were conducted using SAS 9.2 (SAS Institute Inc., Cary, NC, USA) and SPSS statistical analysis software (SPSS Inc., Chicago, IL, USA).

## 3. Results

### 3.1. Histological Findings

Altogether, 30 enlarged glands were found in 24 patients. Twenty patients had a solitary parathyroid adenoma, two patients had double adenomas, and two patients had multiglandular disease. The mean weight of the abnormal parathyroid glands was 677 mg (weight information was not available for four glands).

The postoperative serum Ca values were normalized for all patients. The postoperative iPTH values were normalized for 17 patients. For 7 patients, these values were slightly elevated (ranged from 80 ng/L to 134 ng/L (mean 90 ng/L), but decreased from the preoperative values (ranged from 138 ng/L to 400 ng/L (mean 165 ng/L)). Four glands were visualized in the operation for these patients.

The pathological findings together with the image findings for physician 1 are listed in Table 1.

### 3.2. ^99*m*^Tc-Sestamibi versus ^123^I/^99*m*^Tc-Sestamibi

All image sets with ^123^I/^99m^Tc-sestamibi were significantly more sensitive than any image set with ^99m^Tc-sestamibi, regardless of the acquisition technique (Tables 2 and 3). ^99m^Tc-sestamibi SPECT/CT (image set 4) had the highest specificity (100%), as there were no false-positive readings (Table 2).

**Figure 2:**
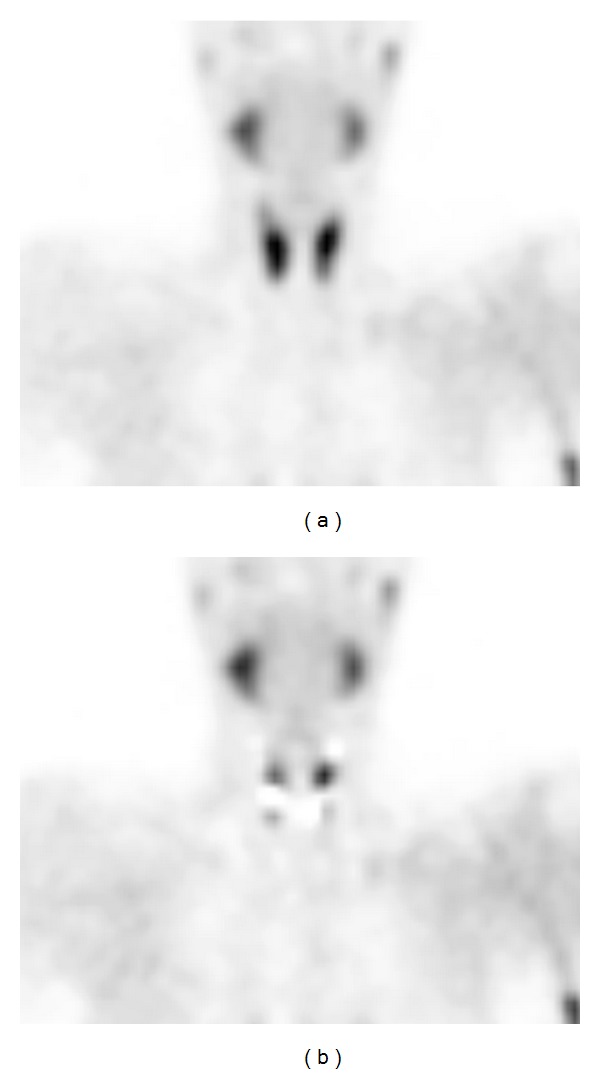
^99m^Tc-sestamibi SPECT (a) and ^123^I/^99m^Tc-sestamibi subtraction SPECT (b) coronal images for a 34-year-old man with secondary hyperparathyroidism. Three hyperplastic parathyroid glands not visualized in coronal ^99m^Tc-sestamibi image are clearly visible in the subtraction SPECT coronal image (b).

^99m^Tc-sestamibi SPECT/CT revealed only 4 abnormal glands, while ^123^I/^99m^Tc-sestamibi subtraction SPECT/CT revealed 23 abnormal glands (Table 1). A representative patient case is shown in Figure 2.

### 3.3. Planar AP with LEHR versus Planar AP with Pinhole versus SPECT/CT

There was no difference in the sensitivity, specificity, or accuracy between the acquisition techniques using ^99m^Tc-sestamibi alone. There was also no difference in the sensitivity, specificity, or accuracy between the acquisition techniques using ^123^I/^99m^Tc-sestamibi (Tables 2 and 3).

**Figure 3:**
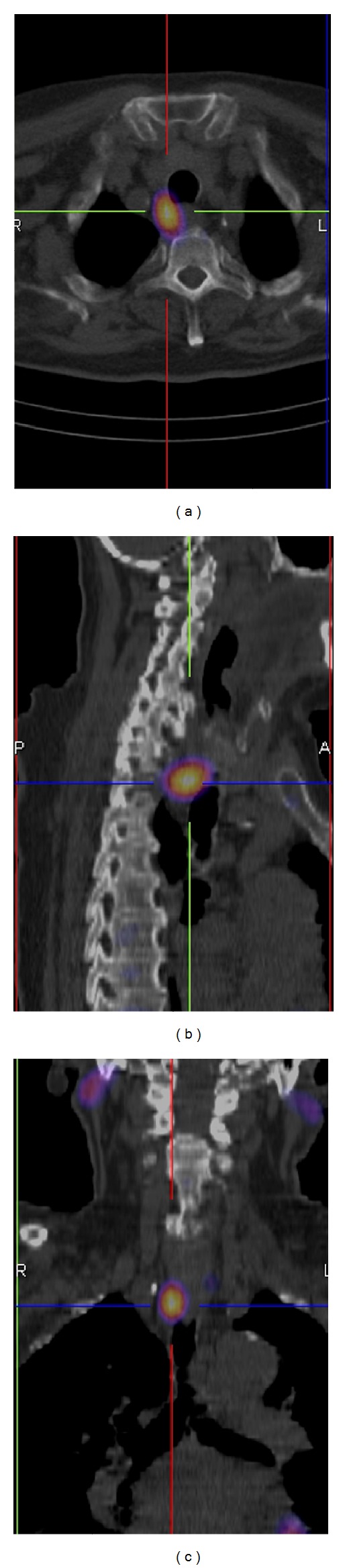
A ^99m^Tc-sestamibi uptake in a parathyroid adenoma located behind the trachea. ^123^I/^99m^Tc-sestamibi subtraction SPECT/CT images (transversal (a), sagittal (b), and coronal (c)).

Although there was no difference in the sensitivity, SPECT/CT may offer invaluable three-dimensional information about the location of the enlarged parathyroid adenomas together with anatomical information (Figure 3).

### 3.4. False-Positive Findings

Ten patients had 12 different false-positive findings (40 false-positive findings if all physicians and image sets are summed up). The false-positive findings are presented in Table 4.

**Figure 4:**
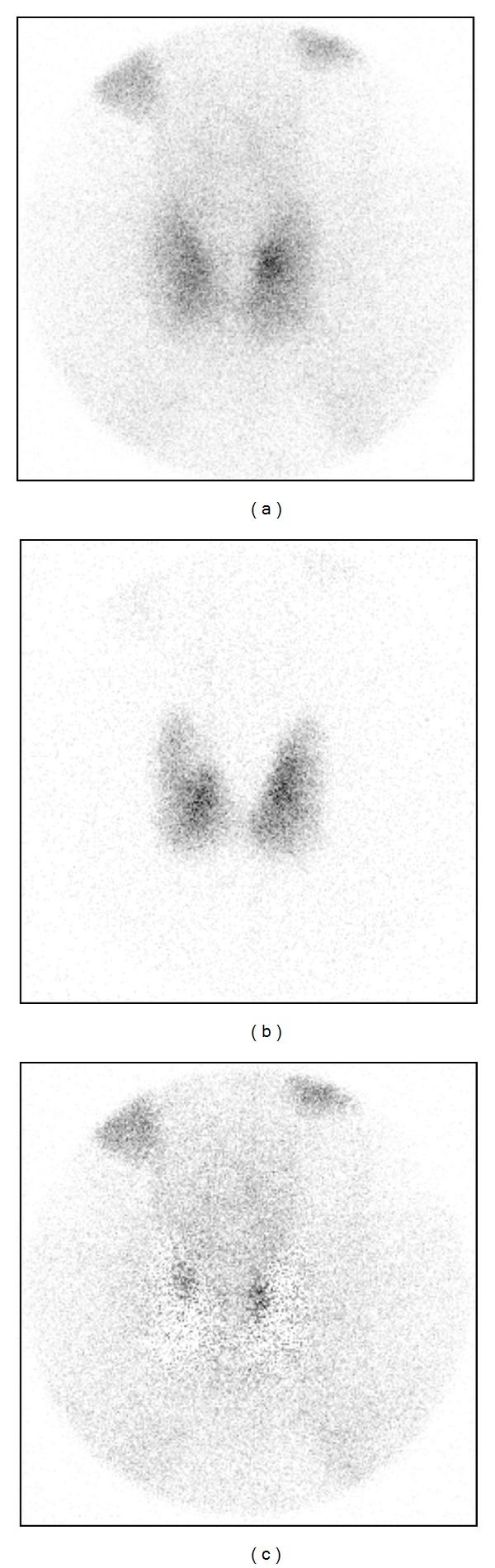
A cold nodule in the upper quadrant of a right thyroid lobe in the ^123^I image (b) causing a false-positive finding in the subtraction image (c).

Four of these were due to cold thyroid nodules in ^123^I images, causing erroneous interpretation in the subtraction image (Figure 4). Uneven ^123^I uptake caused thus 55% of all false-positive findings.

**Figure 5:**
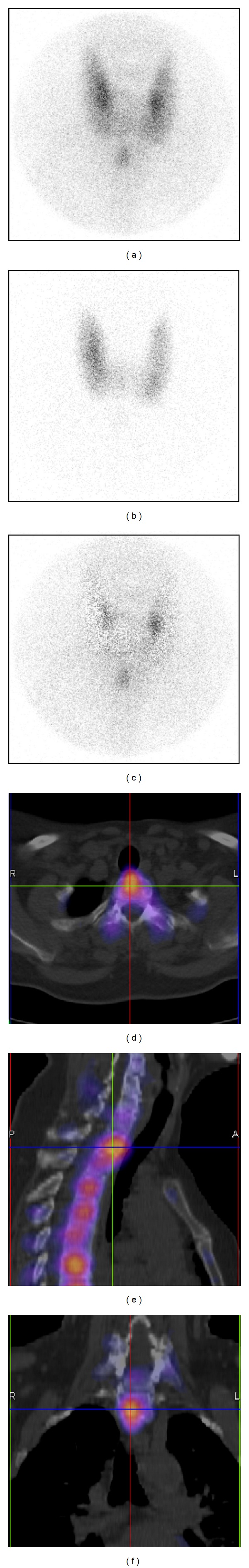
A ^99m^Tc-sestamibi focal uptake inferior to the thyroid seen in the anterior image acquired with a pinhole collimator. ^99m^Tc-sestamibi (a), ^123^I (b), and subtraction image (c). All physicians interpreted this uptake as an abnormal parathyroid gland. The same patient seen in ^123^I/^99m^Tc-sestamibi subtraction SPECT/CT images (transversal (d), sagittal (e), and coronal (f)). Focal uptake was due to bone uptake in the cervical vertebra.

One patient had clear uptake in the ^99m^Tc-sestamibi image below the thyroid as seen in planar images. All physicians interpreted this as a positive finding in the planar images. In the SPECT/CT images, it was revealed that the uptake was in the cervical vertebra (Figure 5). This bone uptake caused 20% of all false-positive findings.

Three false positive findings were due to the “edge effect” in ^123^I/^99m^Tc-sestamibi subtraction SPECT/CT images (residual activity around the thyroid lobes after subtraction). This artefact caused 10% of all false-positive findings.

Four positive findings were caused by an error in image interpretation, mainly in double phase ^99m^Tc-sestamibi images. Difficulty in setting the line between the positive and the negative findings caused 15% of all false-positive findings.

As seen in Table 4, ^123^I/^99m^Tc-sestamibi dual-tracer method with various acquisition techniques produced 90% of all false-positive findings. Subtraction SPECT/CT yielded the lowest percentage of false positives when compared to the other subtraction methods. The double phase ^99m^Tc-sestamibi method produced only 10% of all false-positive findings. The false-positive image rate (%) is presented in Table 5.

### 3.5. False-Negative Findings

There was only one abnormal parathyroid gland (number 10, Table 1) that was visualized in all image sets and by all of the physicians. Thus 23 patients had 29 different false-negative findings (267 false-negative findings if all physicians and image sets are summed up). ^99m^Tc-sestamibi/^123^I subtraction planar images with parallel-hole collimator produced 13.9% of all false-negative findings, ^99m^Tc-sestamibi/^123^I subtraction planar images with pinhole collimator produced 9.7% of all false-negative findings, ^99m^Tc-sestamibi double phase images with parallel-hole collimator produced 22.8% of all false-negative findings, ^99m^Tc-sestamibi SPECT/CT produced 39.3% of all false-negative findings, and ^99m^Tc-sestamibi/^123^I subtraction SPECT/CT produced 14.2% of all false-negative findings (all physicians and all image sets are summed up).

The smallest gland located in this series was 260 mg. There were three smaller abnormal parathyroid glands (160 mg, 170 mg, and 200 mg) that could not be located with any method or imaging technique by any of the physicians. The mean gland size of false-negative and true-positive findings for all physicians and image sets are presented in Table 6 together with the statistical significance.

### 3.6. Interobserver Variability

The *κ* coefficient for the agreement of the results between the four physicians for the five study readings are shown in Table 7.

The highest agreement for accuracy was found for ^99m^Tc-sestamibi SPECT/CT, which did not have any false-positive findings for any physician. The highest agreement for sensitivity was found for the planar subtraction images of ^123^I/^99m^Tc-sestamibi with the pinhole collimator.

## 4. Discussion

Our results clearly show that a dual-tracer method with ^99m^Tc-sestamibi and ^123^I is superior to a single-tracer method with ^99m^Tc-sestamibi for PS, regardless of the acquisition technique used. This has been proposed by other authors as well [4, 9, 13, 14].

To our knowledge, this is the first study comparing planar imaging with parallel-hole and pinhole collimators using the ^123^I/^99m^Tc-sestamibi subtraction method with patients. We could not demonstrate the improved sensitivity from the use of the pinhole collimator that has been shown by several authors when using ^99m^Tc-sestamibi [15–20].

SPECT alone has been shown to improve sensitivity compared with planar imaging with parallel-hole collimators [6, 21–24]. SPECT/CT has been shown to offer precise anatomical localization and an improvement in diagnostic specificity and accuracy over conventional SPECT, especially for patients with previous neck surgery or multiglandular disease [5, 25–32]. The use of SPECT/CT also shortens surgical times (when compared with SPECT alone) and eventually lowers costs [33, 34]. Opposite opinions have also been presented, and the use of SPECT/CT has been found to be important only for locating ectopic parathyroid adenomas [35, 36].

We could not demonstrate an increased sensitivity for ^123^I/^99m^Tc-sestamibi subtraction SPECT/CT when compared with planar ^99m^Tc-sestamibi/^123^I subtraction image sets. However, the use of ^123^I/^99m^Tc-sestamibi SPECT/CT decreased the false-positive rate for three observers when compared with planar ^123^I/^99m^Tc-sestamibi image sets.

The low sensitivity of double phase planar ^99m^Tc-sestamibi or ^99m^Tc-sestamibi SPECT/CT cannot be explained by the rapid washout of ^99m^Tc-sestamibi, as 19 enlarged parathyroid glands were visible in ^123^I/^99m^Tc-sestamibi SPECT/CT images that could not be visualized with ^99m^Tc-sestamibi SPECT/CT. A low sensitivity for a single tracer or the double phase protocols has also been reported by other authors [3, 37].

The low sensitivity of the ^99m^Tc-sestamibi SPECT/CT in this study could not be linked to the timing of the acquisition. SPECT acquisition was started approximately one hour after ^99m^Tc-sestamibi injection. Lavely and coworkers were able to demonstrate much better sensitivity for early-phase SPECT/CT (62%) and also for early planar/delayed planar imaging (56,5%) [5]. The timing of their early planar and SPECT/CT acquisitions was almost identical to ours.

Our results for the ^123^I/^99m^Tc-sestamibi subtraction SPECT/CT are comparable to the results of Neumann and coworkers [26], who demonstrated a sensitivity of 70% and a specificity of 96% in a group of 61 patients with primary hyperparathyroidism. The increase of specificity (when compared with SPECT alone) was explained by reducing the number of false positives.

There were three abnormal parathyroid glands that were visible in ^123^I/^99m^Tc-sestamibi subtraction planar images (with a parallel-hole or a pinhole collimator) but not visible in ^123^I/^99m^Tc-sestamibi subtraction SPECT/CT. This could be due to rapid washout [38] as SPECT/CT was performed one hour later than the planar images were acquired. Thus, the timing of the various acquisitions should be considered carefully, and SPECT/CT should be performed in the early phase so as not to miss abnormal parathyroid gland(s) with rapid washout [10].

The average false-positive rate was comparable to previous reports [6, 11]. In this retrospective study, the five image sets were not reviewed together, which is normally done in our clinical scenario. With careful observation of the ^123^I images of the thyroid, it should be possible to decrease the false-positive rate in subtraction images.

It seems that a major factor influencing detection of abnormal parathyroid glands is their size. The difference of mean gland size of false-negative and true-positive findings was statistically significant for all protocols used in this study.

There was lower number of ectopic glands in this patient group than could be expected [39]. There might have been small ectopic glands which were not recognized in scintigraphy or in surgery. This might explain the slightly elevated iPTH values for 7 patients.

Several imaging protocols for PS with ^99m^Tc-sestamibi are in use, with a wide range of sensitivities (34–100%) reported [8]. No large study exists that compares the accuracy of each [6]. We have shown the superiority of the ^123^I/^99m^Tc-sestamibi subtraction method of PS. The high popularity of the single-tracer method with ^99m^Tc-sestamibi alone can only be explained by its technical simplicity. It is true that the ^123^I/^99m^Tc-sestamibi subtraction method, especially SPECT/CT, is technically demanding. There are several possible sources of artifacts, such as scaling and the subtraction process. In our opinion, the data processing should be performed by an experienced medical physicist.

Even optimal processing of identical ^99m^Tc and ^123^I targets does not give flawless subtraction image, some activity is always left around the edges. In this series, it was in few cases interpreted as a positive finding. To our knowledge, this artefact has not been described earlier concerning parathyroid scintigraphy [40].

The overall interobserver agreement in this study was good. The average *κ* coefficient was 0.79 for accuracy and 0.70 for sensitivity. These results are comparable to previous results [12, 15].

One of the main limitations of this study is the number of patients in the image set 3 (^99m^Tc-sestamibi double phase images). Another limitation of our study relates to the fact that the delay phase was acquired with another gamma camera. However, quality assurance measurements are routinely performed for both cameras. There are no differences in important parameters regarding image quality.

The clinical PS protocol presented in this study, which included various acquisitions, is quite time consuming. The discomfort for the patient should be decreased by rejecting unnecessary acquisitions. This study indicates that the ^123^I/^99m^Tc-sestamibi subtraction method combined with any imaging technique is adequate to locate abnormal parathyroid glands. However, ^123^I/^99m^Tc-sestamibi subtraction SPECT/CT is recommended because it provides accurate three-dimensional information about the location of enlarged parathyroid adenomas together with anatomical information (Figure 3) and may influence the surgical approach [14]. With SPECT/CT, it is also possible to avoid some false-positive findings resulting from the ^99m^Tc-sestamibi uptake in bone structures. The additional use of anterior pinhole images may be useful for recognizing cold thyroid nodules and thus further reducing the false-positive rate. Determining the optimal technical aspects (acquisition and processing parameters, various physical corrections) still requires further study.

## 5. Conclusion

The results of this study show that the ^123^I/^99m^Tc-sestamibi subtraction method combined with any imaging technique is superior for enlarged parathyroid gland localization when compared with ^99m^Tc-sestamibi alone with any acquisition technique. ^123^I/^99m^Tc-sestamibi subtraction SPECT/CT is recommended because it provides accurate three-dimensional information about the location of enlarged parathyroid adenomas. The use of anterior pinhole images may be useful for recognizing cold thyroid nodules and thus reducing the false-positive rate. The overall interobserver agreement for accuracy and for sensitivity in this study was good. Thus the parathyroid scintigraphy is independent of the reporter.

There are two limitations that need to be acknowledged regarding this study. The first limitation is the number of patients in the ^99m^Tc-sestamibi double phase group. Another limitation relates to the fact that the delay phase was acquired with another gamma camera.

## Figures and Tables

**Table 1:** Number of adenomas and hyperplastic glands and image findings for physician 1.

Patient number	Gland number	Weight (mg)	Findings for image set				
			1	2	3	4	5
1	1	170	FN	FN	NA	FN	FN
1	2	570	TP	TP	NA	FN	TP
2	3	980	TP	TP	FN	FN	TP
3	4	830	TP	TP	FN	TP	TP
4	5	1280	TP	TP	FN	FN	TP
4	6	840	TP	FN	FN	FN	TP
4	7	2140	TP	TP	FN	FN	TP
5	8	NA	TP	TP	NA	FN	TP
6	9	1200	TP	TP	NA	FN	TP
7	10	960	TP	TP	TP	TP	TP
8	11	1880	TP	TP	TP	FN	TP
9	12	1160	FN	TP	FN	FN	FN
10	13	299	TP	TP	FN	FN	TP
11	14	200	FN	FN	FN	FN	FN
12	15	260	TP	TP	FN	FN	TP
13	16	570	TP	TP	TP	TP	TP
14	17	370	TP	TP	FN	FN	TP
15	18	300	TP	FN	NA	FN	FN
15	19	400	TP	TP	NA	FN	TP
15	20	NA	TP	TP	NA	FN	TP
16	21	510	TP	TP	NA	FN	TP
17	22	340	TP	TP	FN	FN	TP
18	23	NA	TP	TP	TP	FN	TP
19	24	420	FN	FN	TP	FN	FN
20	25	300	FN	TP	NA	FN	FN
20	26	NA	TP	TP	NA	FN	TP
21	27	520	TP	TP	NA	FN	TP
22	28	400	TP	TP	FN	FN	TP
23	29	550	TP	TP	TP	TP	TP
24	30	160	FN	FN	FN	FN	FN

TP: true positive for abnormal parathyroid gland; FN: false negative for abnormal parathyroid gland; NA: image set not available for patient.

**Table 2:** Sensitivity, specificity, and accuracy for localization of abnormal parathyroid glands.

Image set	Physician	Sensitivity (%)	Specificity (%)	Accuracy (%)
1	1	80.0	93.9	89.6
	2	70.0	95.5	87.5
	3	63.3	97.0	86.5
	4	63.3	97.0	86.5

2	1	80.0	92.4	88.5
	2	80.0	93.9	89.6
	3	76.7	95.5	89.6
	4	76.7	95.5	89.6

3	1	31.6	93.9	76.5
	2	21.1	98.0	76.5
	3	10.5	100.0	75.0
	4	15.8	100.0	76.5

4	1	13.3	100.0	72.9
	2	16.7	100.0	74.0
	3	10.0	100.0	71.9
	4	10.0	100.0	71.9

5	1	76.7	92.4	87.5
	2	76.7	95.5	89.6
	3	56.7	98.5	85.4
	4	63.3	98.5	87.5

**Table 3:** Statistical significance for differences in sensitivity, specificity, and accuracy for comparisons of image sets for physician 1.

Compared image sets	*P* for sensitivity	*P* for specificity	*P* for accuracy
1 versus 2	NS	NS	NS
1 versus 3	0.0117	NS	0.0352
1 versus 4	1.907*E* − 06	0.0455	0.0015
1 versus 5	NS	NS	NS
2 versus 3	0.0117	NS	NS
2 versus 4	1.907*E* − 06	0.0253	0.0041
2 versus 5	NS	NS	NS
3 versus 4	NS	NS	NS
3 versus 5	0.0117	NS	NS
4 versus 5	3.815*E* − 06	0.0253	0.0066

NS: not significant.

**Table 4:** The false-positive findings for all physicians.

Image set	1				2				3				5				Reason for FP	% of FP
Patient number	Physician				Physician				Physician				Physician					
	1	2	3	4	1	2	3	4	1	2	3	4	1	2	3	4
2					RL								RL				Uneven iodine uptake	55
3	RL	RL	RL	RL	RL		RL	RL					RL		RL	RL		
8					RU	RU												
22	RU	RU			RU	RU	RU	RU					RU	RU				

20	RL	RL	RL	RL	RL	RL	RL	RL									Bone uptake	20

4													LL				Edge effect	10
10														LL				
24													RU	RU				

24									LL								Other	15
24	RL					RL			RL									
13										LU								
19									LL									

% of FP	27.5				37.5				10				25					

FP: false positive, RU: right upper, RL: right lower, LU: left upper, and LL: left lower.

**Table 5:** The false-positive image rate (%) for each image set and each physician.

Physician	False-positive rate (%) for image set				
	1	2	3	4	5
1	14.3	17.2	33.3	0.0	17.9
2	12.5	14.3	20.0	0.0	11.5
3	9.5	11.5	0.0	0.0	5.6
4	2.1	3.1	0.0	0.0	1.0

Average	9.6	11.5	13.3	0.0	9.0

**Table 6:** The mean gland size of false-negative and true-positive findings for all physicians and all image sets.

	Image set				
	1	2	3	4	5
Smallest gland visualized	260	260	420	550	260
Mean weight of FN (mg)	300	420	485	420	300
Mean weight of TP (mg)	560	570	960	830	560
*P* (FN versus TP)	0.002	<0.001	0.046	0.026	<0.001

**Table 7:** The comparison of reader agreement (accuracy and sensitivity) between physicians.

*κ* coefficient for	Physicians	Image set				
		1	2	3	4	5
Accuracy	1 versus 4	0.56	0.84	0.67	0.97	0.62
	1 versus 3	0.56	0.84	0.72	0.97	0.56
	1 versus 2	0.69	0.84	0.59	0.92	0.69
	4 versus 3	1.00	1.00	0.96	1.00	0.91
	4 versus 2	0.86	0.78	0.84	0.89	0.59
	3 versus 2	0.86	0.78	0.88	0.89	0.53

Sensitivity	1 versus 4	0.44	0.90	0.30	0.84	0.69
	1 versus 3	0.44	0.90	0.41	0.84	0.57
	1 versus 2	0.56	1.00	0.20	0.61	0.81
	4 versus 3	1.00	1.00	0.77	1.00	0.86
	4 versus 2	0.85	0.90	0.48	0.43	0.69
	3 versus 2	0.85	0.90	0.61	0.43	0.57
